# Report of a Case of Tumor of the Brain

**Published:** 1873-11-01

**Authors:** Norman Bridge


### REPORT OF A CASE OF TUMOR OF THE BRAIN.

READ BEFORE THE CHICAGO MEDICAL SOCIETY, BY NORMAN BRIDGE, M.D., SEPT. 15TH, 1873.

Mrs. M——, wife of a physician, age 51 years, of medium height, had been through life rather delicate in health.

Nine years ago she lost, by death, a son and a daughter, and for several years following was much depressed in consequense. Six years ago she had a condition of despondency, for two or three months, that was so pronounced that she was disinclined to talk, even with her own family. She had no special bodily ailment; was given tonics, and finally recovered.

About five years ago she had several attacks of severe pain in the head, which would continue for three or four days. They seemed to yield to tonic treatment.

For two years previous to her death she had unusual good health. Four months before this sickness began, peculiar electrical phenomena were noted in her body by her husband. Sparks were emitted from her wearing apparel, when taken off at night, on the clothes being shaken and rubbed; also her bedclothes, after arising in the morning, would emit sparks on being shaken.

The sparks were distinctly seen and were emitted as well when the clothes were agitated by the husband as by the patient. This phenomenon ceased soon after the sickness began.

The mother, and three or four of the brothers and sisters of the mother of this patient, have died of apoplexy of the brain.

In September, 1872, she felt unusually well and buoyant. In October she began to feel less vigorous, and complained early in the month of slight deafness in the left ear, which grew worse gradually.

Early in November Dr. S. J. Jones examined her ears, and found some thickening of the tympanum; for this he gave her some treatment, but the symptoms continued to get worse.

About this time (Nov. 1st) pain began to be felt in the left side of the head, far back, which was worse at night, and which grew worse as the hearing grew less; the general strength at the same time gradually failed.

About January 1st, 1873, numbness was noticed on the left side of the face, slight at first, increasing slowly until February 1st, when the whole left side of the head was benumbed, and food or fluid taken into the mouth seemed to the patient as though it was freezing-cold.

The pain becoming all the while more severe, especially at night, it seemed to the patient that something was pressing against the left eye from behind, projecting it forward. About January 1st she complained of a sensation, as though she was falling forward, and when walking would frequently put out her hands to prevent it. This symptom grew slowly worse, until March 1st, when it became unsafe to allow her to attempt walking alone.

Failure of memory was noted about February 1st; and in the beginning of April the reasoning powers began to fail. Her mood was one of great depression, and she had a great tendency to sleep after March 1st; the sleep was unusually sound and heavy, especially on first going to sleep; she would often sleep twelve or fourteen hours in a day.

About June 1st she became talkative and wide-awake, but was pleasant and harmless in her conduct.

By July 10th, the mind became wandering; she fancied somebody was about to shoot her in the head, or shoot some of her family; she failed at times to recognize the members of her family; and in her ravings it became necessary to restrain her.

She had no symptoms of fever during her illness until the latter part of July, when she began to have periodical fever, coming on in the afternoon about 4 o'clock, and continuing until midnight, but not being followed by sweating. On the 27th she fell into a condition of coma, and continued in this state until the 31st, when she died.

The fever during the last two weeks was of a low order of typhoid; but during the last three days there was great heat, and the pulse was 130, and wiry.

During the whole sickness there was severe constipation. No vomitting occurred at any time. The appetite was good until April 1st, when it steadily decreased. The respiration was good throughout; and the pulse showed no mark of irregularity, only a progressing weakness.

There was a dry hacking cough from December 1st, which increased in amount, not in severity, to the end of life.

From Februay 1st, the patient had spells of sneezing daily; she would sneeze two or three times in succession; perhaps a dozen times in the twenty-four hours.

There was no paralysis of any muscles; but by March 1st her inability to guide and manage her feet became apparent, and grew worse continuously. There was a constant tendency for the feet to turn outward, but there was no striking down forcibly of the heels, and the feet could not be managed any better when the patient was looking at them than when she was not. No difficulty was noticed in coordinating the movements of the hands; there was no anæsthesia in any part except the left side of the face and head.

About March 1st the sight of the left eye began to fail, and just before death the sight was nearly lost, and deafness became complete in the left ear.

In February there was a considerable increase in bodily size and weight; but from April 1st there was a gradual emaciation, which, however, was not extreme at the time of death.

The treatment resorted to consisted of alteratives and tonics, from the first; the iodides and hydrargyrum being used in moderate doses; enough of the latter was used at one time early in the case to produce slight ptyalism. Bromides and chloral were given to procure rest and comfort; both worked kindly, and fifteen grains of either being sufficient to quiet the patient.

No opiates were administered; and little effect, save to procure rest, was experienced from any treatment.

A post-mortem examination was made of the brain, Drs. N. S. Davis, Merriman, and the writer, being present, with the following result:

The arachnoid was opaque and thickened, as well as the *pia mater*, over a small space at the vertex, and there were a few small granular bodies, of about the size of millet-seeds, near the falx cerebri. There was no considerable congestion of any of the membranes. No abnormal appearance was found in any part of the cerebrum or its ventricles, except in the choroid plexus, where there was found a few granular bodies, similar in appearance to those found at the vertex.

In the anterior portion of the left lateral hemisphere of the cerebellum, involving the digastric lobe, was found a cystic tumor, with a thick fibrous wall on its posterior aspect, and a thin membranous layer on its anterior aspect.

The whole mass was the size of an English walnut, and the cyst contained about two drachms of slightly opaline fluid.

The whole of the left side of the cerebellum was softened, especially those parts connected with the tumor.

The tumor, as well as the granular bodies, were submitted for examination by the microscope to Dr. I. N. Danforth, who has submitted the following opinion regarding them:

"The large tumor, as well as the smaller masses submitted to me, are typical specimens of small-celled sarcoma. The sac is merely fibrous tissue, not allied to any specific forms of morbid growth."

